# *Trichoderma* application methods differentially affect the tomato growth, rhizomicrobiome, and rhizosphere soil suppressiveness against *Fusarium oxysporum*

**DOI:** 10.3389/fmicb.2024.1366690

**Published:** 2024-02-27

**Authors:** Ananda Y. Bandara, Seogchan Kang

**Affiliations:** Department of Plant Pathology & Environmental Microbiology, The Pennsylvania State University, University Park, PA, United States

**Keywords:** biocontrol, crop growth promotion, *Fusarium*, amplicon metagenomic sequencing, soilborne diseases, suppressive soil, tomato, trichoderma

## Abstract

*Trichoderma* spp. are widely used to enhance crop growth and suppress diverse diseases. However, inconsistent field efficacy remains a major barrier to their use as a reliable alternative to synthetic pesticides. Various strategies have been investigated to enhance the robustness of their application. Here, we evaluated how *T. virens* application methods (pre-, at-, and post-transplant) affect the growth of two tomato varieties and their rhizosphere fungal and bacterial communities. Although the greatest rhizosphere abundance of *T. virens* was observed in the post-transplant application, the at-transplant application promoted tomato growth the most, indicating that greater rhizosphere abundance does not necessarily result in better tomato growth. None of the application methods significantly altered the global rhizosphere fungal and bacterial communities of the tested varieties. Changes in specific microbial genera and guilds may underpin the enhanced tomato growth. We also investigated whether the resulting microbiome changes affect the mycelial growth and conidial germination of *Fusarium oxysporum* f. sp. *lycopersici* and *F. oxysporum* f. sp. *radicis-lycopersici*, soilborne fungal pathogens of tomato, upon exposure to volatile compounds emitted by culturable rhizosphere microbes and metabolites extracted from the rhizosphere soils after *Trichoderma* treatments. Volatile compounds produced by cultured rhizosphere microbes after the at-transplant application suppressed the mycelial growth of both pathogens better than those after the other treatments. Similarly, water-soluble metabolites extracted from the rhizosphere soil samples after the at-transplant application most effectively suppressed the germination rate of *F. oxysporum* spores. Overall, our results suggest that the at-transplant application is most advantageous for promoting the growth of the tested tomato varieties and building soil suppressiveness against the tested fusaria. However, further studies are needed before applying this method to support tomato production. We discuss critical future questions.

## Introduction

Biological control has been widely explored as a method for protecting crop health without disrupting environmental health and ecosystem services. Although many potential biological control agents (BCAs) have been evaluated, approximately 90% of the evaluated fungal BCAs belong to the genus *Trichoderma* (Hermosa et al., 2012; Woo et al., 2014, 2023), with >60% of the globally registered biofungicides containing *Trichoderma* (Verma et al., 2007). There are over 200 described *Trichoderma* species (Bissett et al., 2015; Dou et al., 2020), but only a few have been commercialized as BCAs. *Trichoderma* suppresses pathogens directly through mycoparasitism, competition, and antibiosis and indirectly by inducing plant defense responses (Benítez et al., 2004; Poveda, 2021; Guzmán-Guzmán et al., 2023). Many commercially available *Trichoderma* BCAs are effective against diverse pathogens (Guzmán-Guzmán et al., 2023; Woo et al., 2023). However, their inconsistent field efficacy in different production systems and under varying environmental conditions has restricted their utility as chemical pesticide alternatives. Limited knowledge about which factors cause biocontrol failures and how they work under complex and varied biotic and abiotic conditions has hampered efforts to enhance their effectiveness and design reliable *Trichoderma* deployment strategies (Mazzola and Freilich, 2017; Timmusk et al., 2017). Until reliability and effectiveness are assured, biocontrol will be perceived as a high-risk and high-cost practice.

Various strategies for improving the crop growth promotion and biocontrol efficacies of *Trichoderma* have been reviewed (Fraceto et al., 2018; Ferreira and Musumeci, 2021; Guzmán-Guzmán et al., 2023; Xiao et al., 2023). They include *Trichoderma* strain improvement (Szekeres et al., 2004; Montero-Barrientos et al., 2011; Hassan, 2014), formulation of *Trichoderma* with bioactive compounds (Yang et al., 2011; Nandini et al., 2017; Mbarga et al., 2020), co-application of *Trichoderma* with other beneficial microbes (Rudresh et al., 2005; Shanmugaiah et al., 2009; Mweetwa et al., 2016; Ghorchiani et al., 2018; Karuppiah et al., 2019; Szczałba et al., 2019; Konappa et al., 2020), and combining *Trichoderma* application with organic amendments (Haggag and Saber, 2000; Miranda et al., 2006; Joshi et al., 2009; Yang et al., 2011; Bhadauria et al., 2012; Hannan et al., 2012; Domínguez et al., 2014; Blaya et al., 2016; de Araujo et al., 2019; Sani et al., 2020; Da Silva et al., 2022; Amanullah and Khan, 2023). However, these strategies have their own limitations, calling for continued research to develop highly efficacious biocontrol products and application strategies that are economically viable, technically simple, regulatorily neutral, and easily adoptable.

Delivery methods likely influence the efficacy of BCAs. Generally, plant growth promotion and soilborne disease suppression by *Trichoderma* are achieved through its application to crop growth media (mostly soil) after seedling emergence. The application of *Trichoderma* as a seed treatment is also a promising strategy, especially for crops that are directly drilled into soils (e.g., field/row crops and some horticultural crops), because the soils likely harbor phytopathogenic fungi. The effectiveness of *Trichoderma* seed treatment in promoting crop growth under biotic and abiotic stress conditions has been demonstrated in field crops like pea (Harman et al., 1980), cotton (Hanson, 2000), wheat (Xue et al., 2017), cowpea (Adekunle et al., 2001), common bean (Carvalho et al., 2014), and corn (Estévez-Geffriaud et al., 2020). When directly planted in field soils, *Trichoderma* seed treatment alleviated biotic and abiotic stresses in tomatoes (Mastouri et al., 2010). Even when grown in autoclaved potting mix (e.g., sand and vermicompost) in the absence of any pathogens, *T. asperellum* seed treatment significantly enhanced the growth of tomato, brinjal, chili, okra, ridge gourd, and guar (Singh et al., 2016). However, in commercial production systems, most high-value brassicaceous (e.g., broccoli, Brussels sprouts) and solanaceous (e.g., tomato, pepper) vegetables are typically transplanted into the soil after raising them using autoclaved potting mixes in nursery trays. Therefore, priming the root system with *Trichoderma* during seedling growth could enhance seedling vigor and better protect the seedlings from soilborne diseases after transplantation.

Rapid advances in analyzing the composition and changes of plant-associated microbiomes under diverse conditions through massive sequencing of phylogenetically informative loci have uncovered how rhizosphere microbiomes affect crop yield, quality, and tolerance to biotic and abiotic stressors (Berendsen et al., 2012; Chaparro et al., 2012; Berg et al., 2017; Kwak et al., 2018; Zhou et al., 2019). Given their critical roles in plant growth and health, judicious manipulation of rhizosphere/bulk soil microbiomes is a promising approach for achieving greener agriculture. The effectiveness of *Trichoderma*-based biocontrol can be improved through a better understanding of how its introduction results in structural and functional changes in the soil microbiome and how the resulting changes affect crop growth and the suppressiveness of soilborne pathogens. We aimed to advance this understanding by initially assessing how the application method (collectively characterized by the time of application, the substrate to which the application is administered, and the duration of *T. virens*-tomato root system contact; see Materials and Methods for details) of *T. virens* affects the growth of two tomato varieties and subsequently analyzing how each application method affects the tomato rhizosphere fungal and bacterial community structure using amplicon metagenomic sequencing (metabarcoding). *Trichoderma* suppresses pathogens by secreting antifungal metabolites, including volatile compounds (Li et al., 2018; Woo et al., 2023). However, how antimicrobial compounds produced by soil microbes affect *Trichoderma* and pathogens remains largely unknown. We evaluated how volatile compounds emitted by culturable rhizosphere microbes of two tomato varieties after individual *Trichoderma* treatments affect the mycelial growth of *T. virens* and two soilborne fungal pathogens of tomatoes, *F. oxysporum* f. sp. *lycopersici* (Fusarium wilt) and *F. oxysporum* f. sp. *radicis-lycopersici* (Fusarium crown and root rot). They are among the most problematic *Fusarium* pathogens for tomato production (McGovern, 2015). We also assessed how water-soluble metabolites extracted from the rhizosphere soils of the two tomato varieties after different *Trichoderma* treatments affected the germination rate of *T. virens* and *F. oxysporum* conidia.

## Materials and methods

### Growing tomato plants, preparation and application of *Trichoderma* inoculum, measuring tomato growth, and collecting rhizosphere soils

Seeds of the tomato varieties Bonny Best (Fusarium wilt susceptible) and Red Deuce (Fusarium wilt resistant) were planted in seed starter trays filled with autoclaved (121°C and 20 psi for 30 min) Premier Horticulture 10380RG Pro-Mix Professional Grower Mix (Premier Horticulture Inc., Canada). Starter trays were kept on bottom watering trays and maintained in a growth chamber at 25°C, 75% RH, and 16 h light at 100 μmol/8 h dark.

The *T. virens* isolate used by Li et al. (2018) was cultured on PDA at 27°C for 1 week to harvest conidia using sterile distilled water. Conidial suspension was passed through four layers of sterile cheesecloth placed in sterile 50 mL Falcon tubes. After centrifuging the tubes at 4,000 rpm at 4°C for 10 min, the supernatant was discarded. The conidial concentration was determined using a hemocytometer after resuspension in sterile distilled water. The resulting conidial suspension was used for the following treatments: T1 (*T. virens* conidia in the bottom watering tray for a duration of 1 week prior to transplant, after introducing the conidia at 2 weeks after planting the seeds), T2 (*T. virens* conidia in the bottom watering tray for a duration of 30 min prior to transplantation, after introducing the conidia at 3 weeks after planting the seeds), T3 (*T. virens* conidia applied to the soil 1 week post-transplantation, after 3 weeks of pre-transplant growth upon planting the seeds in the bottom watering tray containing sterile distilled water), and CON (control treatment with sterile distilled water in the bottom watering tray for a duration of 3 weeks prior to transplant, after planting the seeds). Note that the T1, T2, and T3 treatments are hereafter referred to as pre-, at-, and post-transplant treatments/applications. Seedlings from all treatments were transplanted 3 weeks after planting seeds. The final conidial concentration was 10^6^ conidia/mL of water for T1 and T2, and 10^6^ conidia/cm^3^ of soil for T3. A batch of topsoil collected from the Penn State Russell E. Larson Agricultural Research Center at Rock Springs was used for transplantation. Forty-eight quart-sized Ziploc bags were filled with thoroughly homogenized soil. Seedlings were transplanted into bags placed in 3-inch plastic nursery pots. The pots were maintained in a growth chamber at 25°C, 75% RH, and 16 h light under a 100 μmol/8 h dark cycle. The amounts of water and fertilizers applied after transplantation were kept consistent across the treatments.

The plants were uprooted at 11 weeks after transplantation, and their rhizosphere soils (soil up to 2 mm surrounding the root) were collected in sterile 15 mL Falcon tubes. The tubes were stored at −80°C until genomic DNA extraction and other analyses. Fresh weights of the shoots and soil-free roots of each plant were measured. The root/shoot ratio of each plant was calculated. The treatment structure of the experiment was “two-factor factorial,” where the first factor was “*Trichoderma* treatment” with four levels (CON, T1, T2, and T3) and the second factor was “tomato variety” with two levels (Bonny Best and Red Deuce). Six plants/pots (replicates) were used for each *Trichoderma* treatment-tomato variety combination. The treated pots were randomly arranged in the growth chamber. Hence, the design structure of the experiment was completely randomized. The experiment was repeated one time.

### Statistical analysis of the tomato growth traits

To test the main/simple effects of *Trichoderma* treatment and tomato variety on the tomato shoot and root weights, ANOVA was conducted using the PROC GLIMMIX procedure in SAS (version 9.4, SAS Institute, Cary, NC) at the 5% significance level (*α* = 0.05). Initially, the data from two repetitions were analyzed separately. As similar results were observed, the final analysis was performed using the average values of the two repetitions. As shoot and root weights are continuous measurements, statistical modeling was performed with the normal/Gaussian distribution with the “Identity” link function. The restricted maximum likelihood (REML) method was used to compute the variance components. Newton–Raphson with Ridging was used as the non-linear parameter optimization method. The assumptions of identical and independent (homogenous) distribution of residuals were tested using studentized residual plots and Brown and Forsythe’s test. The assumption of normal distribution of residuals was checked using *Q*–*Q* plots and the Shapiro–Wilk test. If residuals were found to be non-homogenously distributed (showing heteroskedasticity), appropriate heterogeneous variance models were fitted by specifying a “*random residual/group* = …” statement (where group = *Trichoderma* treatment or tomato variety). The computation of the degrees of freedom for the denominator of *F*-tests and the adjustment of the standard errors of fixed effects were performed using the Kenward-Roger option. Between the two heterogeneous variance models, the model with the lowest Bayesian Information Criterion (BIC) was selected as the best-fitting model. As the computed root/shoot ratios are proportions (between 0 and 1), the data were modeled with the beta distribution along with the “Logit” link function. The generalized linear mixed model (GLMM) estimation was based on integral approximations to the likelihood, where Laplace was used as the likelihood approximation method. The maximum likelihood (ML) method was used to compute variance components. The Newton–Raphson with Ridging method was used as the non-linear parameter optimization method. The degrees of freedom method was residual. The *inverse link* function was used to create the means and associated standard errors at the data scale. The mean separation (all pairwise comparisons of the levels of the factor “*Trichoderma* treatment”) was performed with adjustments for multiple comparisons using the Tukey–Kramer test at 5% significance level/experimental-wise error rate (*α*_EER_ = 0.05).

### DNA extraction, polymerase chain reaction, sequencing, sequence data processing, and analysis

DNA was extracted from 0.5 g of thoroughly homogenized rhizosphere soil using the FastDNA^™^ SPIN Kit for Soil (MP Biomedicals, Irvine, CA, United States). The concentration of genomic DNA was determined using the Qubit dsDNA BR Assay Kit and 1% agarose gel electrophoresis. PCR amplification of the rRNA ITS1 region was performed using ITS1F (5′-CTTGGTCATTTAGAG GAAGTAA-3′) and ITS2R (5′-GCTGCGTTCTTCATCGATGC-3′) primers. PCR amplification of the 16S rRNA V3–V4 region was performed using 338F (5′-ACTCCTACGGGAGGCAGCAG-3′) and 806R (5′-GGACTACHVGGGTWTCTAAT-3′) primers. PCR, preparation of amplicon libraries, and sequencing were performed by the Beijing Genomic Institute (BGI). Sequencing was performed using the PE300 Illumina MiSeq platform.

Initial sequence data processing was performed in R (v.4.2.1) using the DADA2 (v.1.26) pipeline (Callahan et al., 2016). Primer sequences were removed from reads using cutadapt command-line version 2.8 (Martin, 2011), a plugin in DADA2. Fungal taxonomic assignment was performed using the UNITE General FASTA release (v.9) database (Kõljalg et al., 2005). Silva database (v.138.1) was used to assign taxonomy for bacteria (Quast et al., 2012). DADA2 outputs were converted into phyloseq objects using the phyloseq R package (v.1.42) (McMurdie and Holmes, 2013) and the Biostrings R package (v.2.66) (Pagès et al., 2022). These phyloseq objects were used as the source for downstream global and specific analyses. Following the standard procedure, all global analyses, including beta diversity, alpha diversity, and relative abundance (see below), were performed in R (v.4.2.1) using rarefied read counts (based on the minimum available reads per sample among all samples; fungi = 78,869, bacteria = 41,591). In addition, the rarefied read counts were converted into proportions to perform beta diversity and relative abundance analyses.

To analyze the *β*-diversity, the ordinate function of the phyloseq R package (v.1.42) (McMurdie and Holmes, 2013) was used with distance = bray and method = PCoA specifications. PCoA plots were visualized using the ggplot2 R package (v.3.3.3) (Wickham, 2016). A distance matrix was generated using the phyloseq::distance function of the phyloseq R package (v.1.42), with Bray–Curtis specification. The adonis2 function of the Vegan R package (v.2.6.4) (Oksanen et al., 2022) was applied to the Bray–Curtis distance matrix to perform a global scale permutational multivariate analysis of variance (PERMANOVA) to test the significance of the effect of *Trichoderma* treatment and tomato variety on the community composition dissimilarity between samples. A global scale multivariate homogeneity of group dispersions (variances) was performed using the betadisper and permutest functions of the Vegan R package (v.2.6.4) to test the null hypothesis of homogenous dispersions of samples belonging to different *Trichoderma* treatments and tomato varieties. The pairwise.adonis function of the pairwiseAdonis R package (v. 0.4) (Martinez Arbizu, 2020) was used as a *post hoc* test of the global PERMANOVA to assess which pairs of *Trichoderma* treatments have significantly different centroids. Similarly, the permutest function with pairwise = true specification was used as a *post hoc* test of the global beta dispersion test to assess which pairs of *Trichoderma* treatments have homogenous dispersions. The community composition dissimilarity between samples belonging to a given pair of *Trichoderma* treatment (and for two levels of tomato variety) levels was declared significant when the adjusted *p*-value obtained through pairwise.adonis test was smaller than 0.05, and the adjusted *p*-value obtained through the pairwise beta dispersion test was larger than 0.05. In both the global and pairwise analyses, significance was assessed using 999 permutations.

The alpha diversity in each sample was estimated using the microbiome R package (v.1.20.0) (Lahti and Shetty, 2023) with the following indicators: Chao1 richness, Shannon diversity, Coverage diversity (=number of species needed to cover 50% of the ecosystem), Pielou evenness, Core abundance (=relative proportion of the core species that exceed detection level 0.1% in over 50% of the samples), and Rare abundance (complement of the core abundance). Subsequently, ANOVA was performed using the GLIMMIX procedure in SAS (v.9.4) to test the main/simple effects (*α* = 0.05) of *Trichoderma* treatment and tomato variety on the estimated alpha diversity indicators. Because Pielou evenness, Core abundance, and Rare abundance values are proportions, data from those three indicators were modeled with the beta distribution. The Shannon diversity values of each sample were converted into proportions by dividing them by the largest Shannon diversity value in the dataset. Modified Shannon diversity values were also modeled using the beta distribution. As Caho1 richness and Coverage diversity values are counts, modeling was performed with a negative binomial distribution. In all cases, the remaining model specifications were identical to those used for the statistical analysis of root/shoot ratios.

The relative abundances of taxonomic groups at the phylum and genus levels were determined using the *transform_sample_counts* function in the R package *phyloseq*. The relative abundance of top fungal phyla/genera was depicted as stack bar plots using the *ggplot()* function in the R package ggplot2.

ANOVA was performed using the GLIMMIX procedure to test the main/simple effects (*α* = 0.05) of *Trichoderma* treatment and tomato variety on the proportional abundance (=ratio between “sum” of the “non-rarefied” read counts of a species/genus/guild of interest in a given rhizosphere sample and total read count of the same sample) of *T. virens*, beneficial fungi (*Acremonium*, *Cadophora*, *Chaetomium*, *Clonostachys*, *Mortierella*, *Paraphaeosphaeria*, *Penicillium*, *Syncephalis*, *Humicola*, *Marquandomyces*, *Metacordyceps*, *Metarhizium*, *Linnemannia*), *Fusarium*, other fungal phytopathogens (*Albifimbria*, *Aspergillus*, *Cladosporium*, *Dactylonectria*, *Didymella*, *Gibellulopsis*, *Melanconiella*, *Microdochium*, *Mycoleptodiscus*, *Neocosmospora*, *Neonectria*, *Paecilomyces*, *Plectosphaerella*, *Pseudopithomyces*, *Rhizoctonia*, *Talaromyces*), bacterial biocontrol agents (*Arthrobacter*, *Bdellovibrio*, *Flavisolibacter*, *Lysobacter*, *Massilia*), bacterial plant growth promoters (*Devosia*, *Mucilaginibacter*), nitrifiers (*Chujaibacter*, *MND1*, *Nitrolancea*, *Nitrosospira*, *Nitrospira*, *Pseudarthrobacter*, *Pseudolabrys*), symbiotic nitrogen fixers (*Allorhizobium-Neorhizobium-Pararhizobium-Rhizobium*, *Bradyrhizobium*, *Mesorhizobium*), free-lining nitrogen fixers (*Bacillus*, *Clostridium*, *Paenibacillus*, *Rhodopseudomonas*), soil Polycyclic Aromatic Hydrocarbon/toxin degraders (*Mycobacterium*, *Sphingomonas*), phytopathogens (*Burkholderia-Caballeronia-Paraburkholderia*, *Ralstonia*, *Streptomyces*), and denitrifiers (*Burkholderia-Caballeronia-Paraburkholderia*, *Conexibacter*, *Rhodanobacter*). Modeling was performed using the beta distribution, and the model specifications were identical to those used for the statistical analysis of root/shoot ratios. We defined a guild as a group of genera that can potentially contribute to a specific ecological function/service (e.g., symbiotic nitrogen fixation). By using proportional abundance instead of absolute abundance, we standardized the effect of varying numbers of total read counts among samples (different library sizes) on the abundance of the species/genus/guild of interest in the respective samples. Furthermore, the use of “non-rarefied” read counts as the numerator is computationally and ecologically more realistic and sensical than computing the proportional abundance using “rarefied” read counts (=ratio between the sum of the “rarefied” read counts of a species/genus/guild in a given sample and the total number of read counts of the “smallest” sample), as rarefaction can artificially inflate or deflate the abundance of taxa as a result of the random resampling. Note that if proportional abundance is computed using “rarefied” read counts, the total read count number of the smallest sample should be used as the common denominator when rarefaction is performed based on the “minimum” read count sample among “all” samples, as rarefaction can be performed at any preferred number at the cost of removing samples from the data set or a given analysis.

### Determination of whether volatiles emitted by cultured rhizosphere microbes after different *Trichoderma* treatments affect the *in-vitro* growth of *T. virens*, *F. oxysporum* f. sp. *lycopersici*, and *F. oxysporum* f. sp. *radicis*-*lycopersici*

We used a modified version of the sandwiched plate assay described in Li et al. (2018). First, X plates (plates with four sections) were prepared using four growth media, including R2A, tryptic soy agar (TSA), potato dextrose agar (PDA), and malt extract agar (MEA). Each plate contained all four media without any antibiotics or antifungal compounds. R2A/TSA and PDA/MEA were used to maximize the culturable fraction of bacteria and filamentous fungi/yeasts, respectively. Second, 2.000 g of each rhizosphere soil sample was homogenized in 10 mL of sterile distilled water, and 5 μL of each soil suspension was spread onto each section of the plate using sterile glass beads. The inoculated plates were incubated at 27.5°C for 2 days (dense colonies appeared at this time). After incubating PDA plates inoculated with mycelial plugs of *T. virens*, *F. oxysporum* f. sp. *lycopersici*, and *F. oxysporum* f. sp. *radicis-lycopersici* for 24 h, these plates were sandwiched with 2 days-old X plates containing rhizosphere microbial colonies and incubated at 27.5°C for 3 days before measuring the fungal colony diameters. This experiment was conducted two times using the soil samples collected after the two growth chamber experiments (see above). To test the main/simple effects of rhizomicrobial volatiles from *Trichoderma* treatment and tomato variety on the fungal colony diameters, ANOVA was performed using the PROC GLIMMIX procedure in SAS (v.9.4, SAS Institute, Cary, NC) at the 5% significance level (*α* = 0.05). Initially, the data from two repetitions were analyzed separately. However, the final analysis was performed using the average values of two repetitions because the results of the separate analyses were interpretationally identical. Model specifications and the method of mean separation were identical to those used for the statistical analysis of tomato shoot and root weights (see above).

### Determination of whether water-soluble metabolites extracted from tomato rhizosphere soils after receiving different *Trichoderma* treatments affect the germination of *T. virens*, *F. oxysporum* f. sp. *lycopersici*, and *F. oxysporum* f. sp. *radicis-lycopersici* conidia

The original soil suspensions (*n* = 48) prepared for sandwich assays (see above) by suspending 2.000 g of soil in 10 mL of sterile distilled water were thoroughly vortexed and centrifuged for 10 min at 4,000 rpm. The supernatants were filtered using 0.2 μM filters to obtain sterile metabolite extracts. The ability of these metabolite extracts to modulate the conidial germination rate of *T. virens* and *F. oxysporum* was assayed under *in vitro* conditions using a redox reagent, Alamar Blue (AB: Thermo Fisher Scientific). As AB is an indicator of the cellular metabolic activity, it can be used for high-throughput determination of conidial viability and germination rate. AB, in its oxidized form, is blue and non-fluorescent. Germinating conidia cause a chemical reduction of AB from non-fluorescent blue to fluorescent red. Since AB reduction is directly proportional to the conidial germination rate, the effects of soil metabolites on the germination rate can be measured by monitoring the level of AB reduction indicated by the resulting absorbance changes.

For the assay, conidial suspensions of three fungi were prepared using pH-neutral potato dextrose broth (PDB). The assay was set up on a round bottom 96-well plate. One hundred  μL of PDB containing 10^5^ conidia/ml, 10 μL of AB, and 10 μL of metabolite extract were mixed in each well. For each fungus, 48 reaction wells (48 metabolite extracts = four treatments, two varieties, and six replicates) were used. The wells for negative controls contained the same volumes of PDB, AB, and metabolite extract. Plates were incubated at 25°C for 24 h before measuring the absorbance at 570 and 600 nm using a plate reader. AB reduction (proportional) in each well was computed using the following equation:


$$\mathrm{AB}\;\mathrm{reduction}=\frac{\left(O2\times A1\right)-\left(O1\times A2\right)}{\left(R1\times N2\right)-\left(R2\times N1\right)}$$

where.

O1 = molar extinction coefficient of oxidized AB at 570 nm (=80,586); O2 = molar extinction coefficient of oxidized AB at 600 nm (=117,216); R1 = molar extinction coefficient of reduced AB at 570 nm (=155,677); R2 = molar extinction coefficient of reduced AB at 600 nm (=14,652); A1 = absorbance value of test wells at 570 nm; A2 = absorbance value of test wells at 600 nm; N1 = absorbance value of negative control well at 570 nm; and N2 = absorbance value of negative control well at 600 nm.

The experiment was conducted two times using soil samples collected from the two growth chamber experiments (see above). To test the effects of soil metabolites resulting from *Trichoderma* treatments and tomato varieties on the germination rate of conidia (=AB reduction), ANOVA was conducted using the PROC GLIMMIX procedure in SAS (v.9.4) at the 5% significance level (*α* = 0.05). Initially, the data from two repetitions were analyzed separately. As the results of the separate analyses were interpretationally identical, the final analysis was performed using the average values of two repetitions. Model specifications and the method of mean separation were identical to those used for the statistical analysis of the tomato root/shoot weight ratio described above.

## Results

### Effect of *Trichoderma* treatment, tomato variety, and their interaction on tomato growth

**Figure 1:**
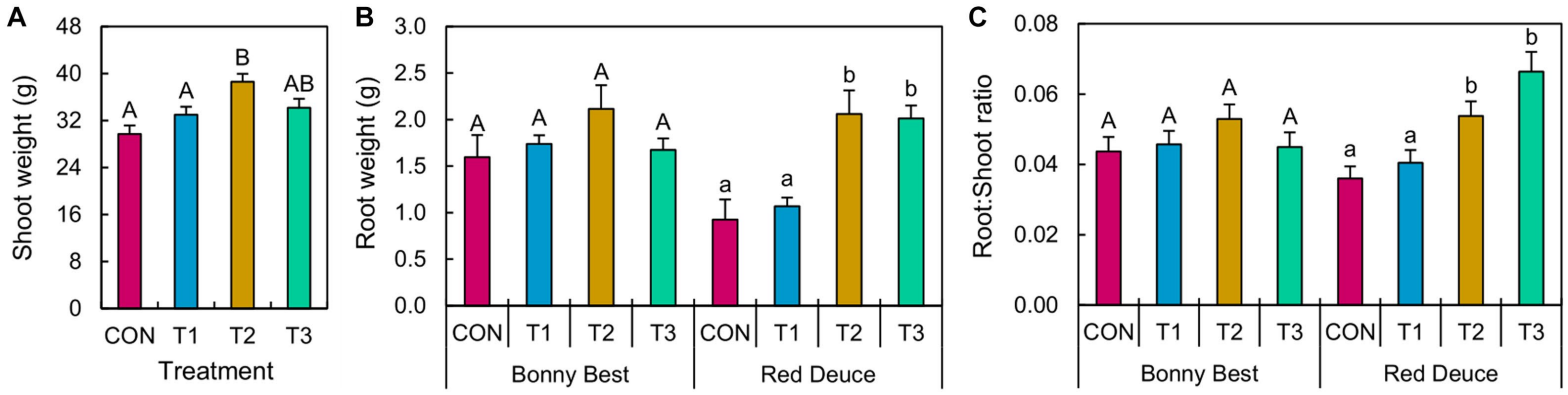
Effects of *Trichoderma* application methods on tomato growth. Mean **(A)** shoot weight, **(B)** root weight, and **(C)** root-to-shoot ratio of the tomato plants treated with CON (control treatment), T1 (pre-transplant treatment with *T. virens*), T2 (at-transplant treatment with *T. virens*), and T3 (post-transplant treatment with *T. virens*) are shown. Means followed by the same letters within each letter type (upper or lower case) are not significantly different at the 5% level (=5% experimental-wise error rate) of significance after adjusting the *p*-values for multiple comparisons using the Tukey test. Error bars represent standard errors. **(A)** shows the main effect of *Trichoderma* treatment, while **(B)** and **(C)** show its simple effects.

ANOVA revealed a significant main effect of *Trichoderma* treatment on shoot weight and a significant *Trichoderma* treatment × tomato variety interaction effect on root weight and the root/shoot ratio (Supplementary Table S1). T2 (at-transplant application) significantly improved shoot weight compared to T1 (pre-transplant application) and CON (Figure 1). Both T2 and T3 (post-transplant application) increased the root weight and root/shoot ratio of Red Deuce compared to CON and T1 (Figure 1). However, none of the *Trichoderma* treatments did significantly better than others in enhancing the root weight or root/shoot ratio of Bonny Best (Figure 1).

### Effect of *Trichoderma* treatment, tomato variety, and their interaction on alpha diversity

The *Trichoderma* treatment × tomato variety interaction was significant (*α* = 0.05) for the fungal and bacterial Shannon diversity and fungal Pielou evenness, whereas the main effect of *Trichoderma* treatment was significant for the fungal core and rare abundances (Supplementary Table S2). The mean fungal and bacterial Shannon diversities were not significantly different among the four treatments in Red Duce (Supplementary Figures S1, S2). In Bonny Best, the fungal Shannon diversities of T1 and T2 were greater than those of CON and T3 (Supplementary Figure S1), whereas T1 increased the bacterial Shannon diversity compared to CON, T2, and T3 (Supplementary Figure S2). The mean fungal Pielou evenness among the treatments was not significantly different in Red Duce. However, in Bonny Best, T1 significantly increased the evenness compared to CON and T3 (Supplementary Figure S1). T3 decreased the fungal core abundance compared to the other *Trichoderma* treatments across both varieties, whereas T1 decreased the fungal core abundance compared to CON (Supplementary Figure S1). T3 increased the fungal rare abundance compared to the other treatments across both varieties, and T2 increased the abundance compared to CON (Supplementary Figure S1).

### Effect of *Trichoderma* treatment and tomato variety on the rhizosphere fungal and bacterial community composition dissimilarity (beta diversity)

**Figure 2:**
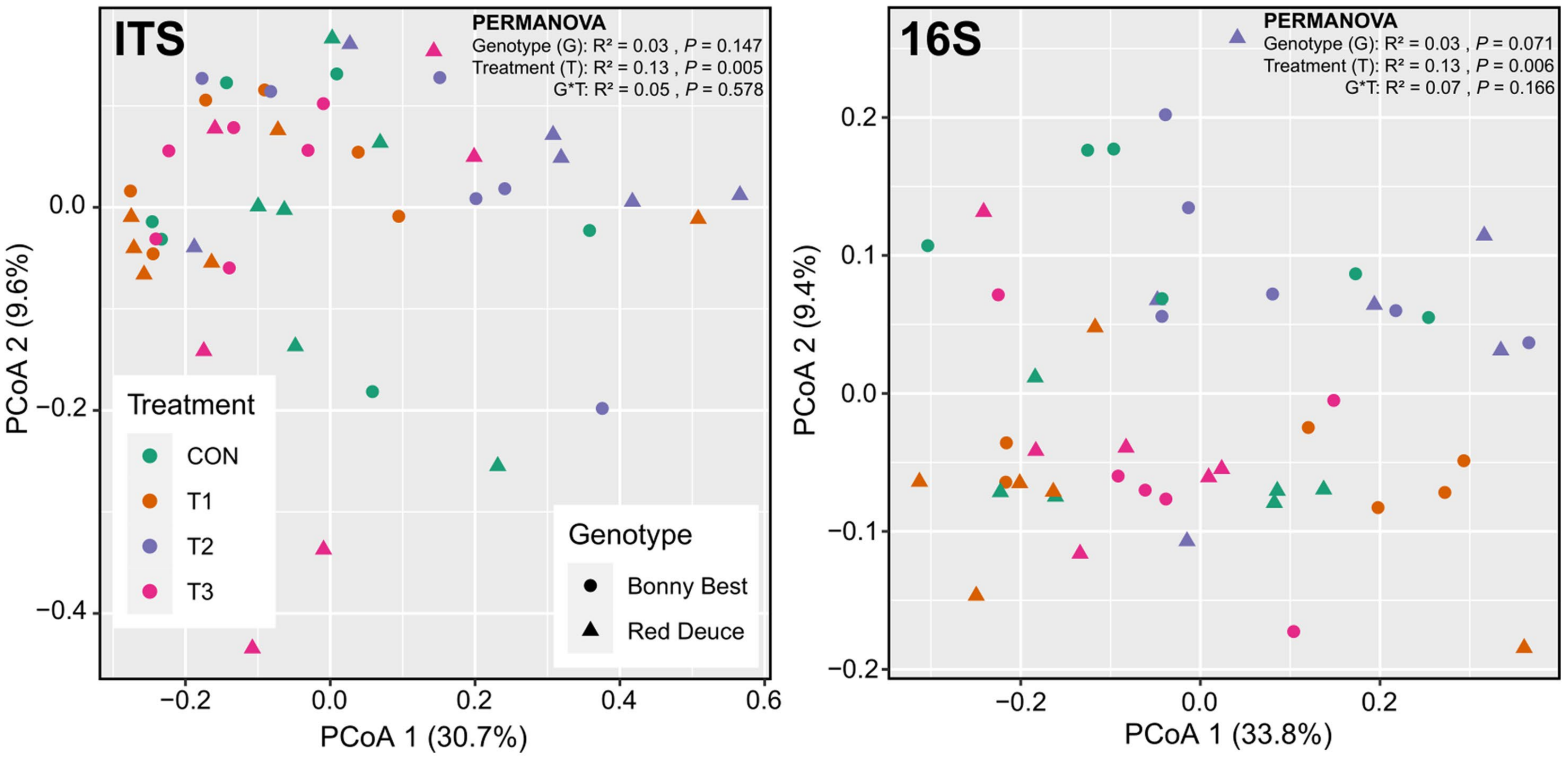
Principal coordinate analysis (PCoA) plots showing the Bray–Curtis dissimilarity-based distribution pattern of rhizosphere soil samples for fungal and bacterial communities. Samples belong to CON (control treatment), T1 (pre-transplant treatment with *T. virens*), T2 (at-transplant treatment with *T. virens*), and T3 (post-transplant treatment with *T. virens*), and two tomato varieties (Bonny Best and Red Deuce) are depicted. Marker color and shape correspond to the *Trichoderma* treatment and tomato variety, respectively.

According to the principal coordinate analysis (PCoA), the first two principal coordinates explained 40.5 and 43.5% of the total variation for fungi and bacteria, respectively (Figure 2). The global PERMANOVA revealed a significant *Trichoderma* treatment effect on fungal and bacterial beta diversities, but the effects of tomato variety and its interaction with *Trichoderma* treatment were non-significant (Figure 2). Per pairwise PERMANOVA, both fungal and bacterial community compositions were significantly dissimilar between T2 and T3 (Supplementary Table S3). This finding was further supported by the non-significant beta dispersion between T2 and T3 (Supplementary Table S3). None of the *Trichoderma* treatments significantly altered the fungal or bacterial community composition compared to CON at the global scale (Supplementary Table S3).

### Effects of *Trichoderma* treatments and tomato varieties on the relative abundance of major fungal and bacterial phyla and genera

**Figure 3:**
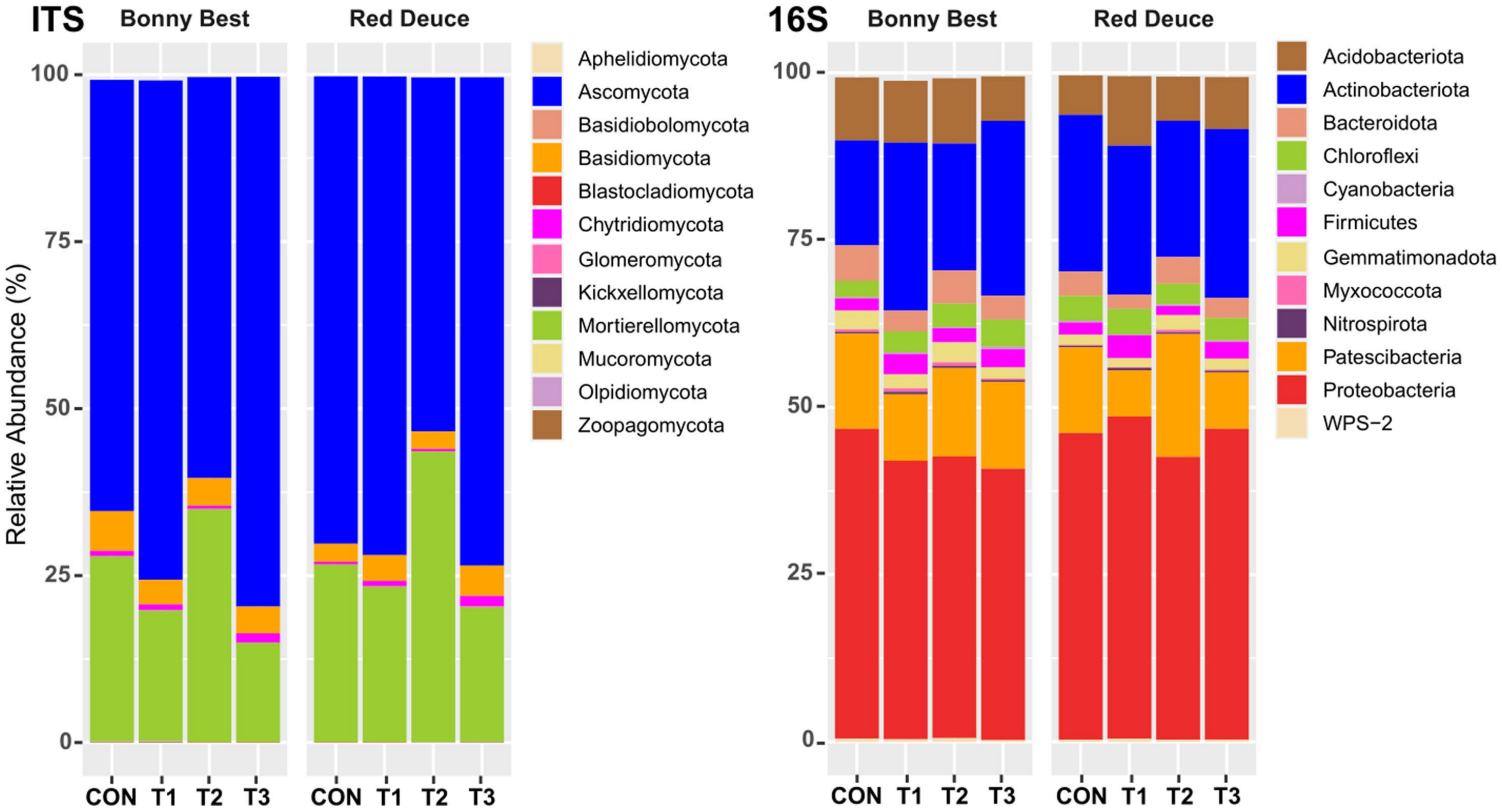
Effect of *Trichoderma* treatment on the mean relative abundance of the top 12 fungal and bacterial phyla. Treatments include CON (control treatment), T1 (pre-transplant treatment with *T. virens*), T2 (at-transplant treatment with *T. virens*), and T3 (post-transplant treatment with *T. virens*).

All *Trichoderma* treatments altered the relative abundance of fungal phyla such as Ascomycota, Basidiomycota, Chytridiomycota, and Mortierellomycota, but the nature of alteration varied among the treatments (Figure 3). In both tomato varieties, the abundance of Ascomycota was highest in T3, followed by T1, CON, and T2. The opposite was observed for Mortierellomycota. T3 resulted in the highest abundance of Chytridiomycota in both varieties, whereas T2 caused the lowest. All *Trichoderma* treatments decreased the abundance of Basidiomycota compared to CON in Bonny Best, whereas the opposite was true in Red Deuce. For bacterial phyla, all *Trichoderma* treatments markedly decreased the abundance of Bacteroidota, Patescibacteria, and Proteobacteria compared to CON in Bonny Best, while increasing the abundance of Actinobacteriota, Chloroflexi, and Firmicutes (Figure 3). In Red Deuce, T1 resulted in the highest abundance of Acidobacteriota, Chloroflexi, Firmicutes, and Proteobacteria and the lowest abundance of Bacteroidota, Gemmatimonadota, and Patescibacteria.

**Figure 4:**
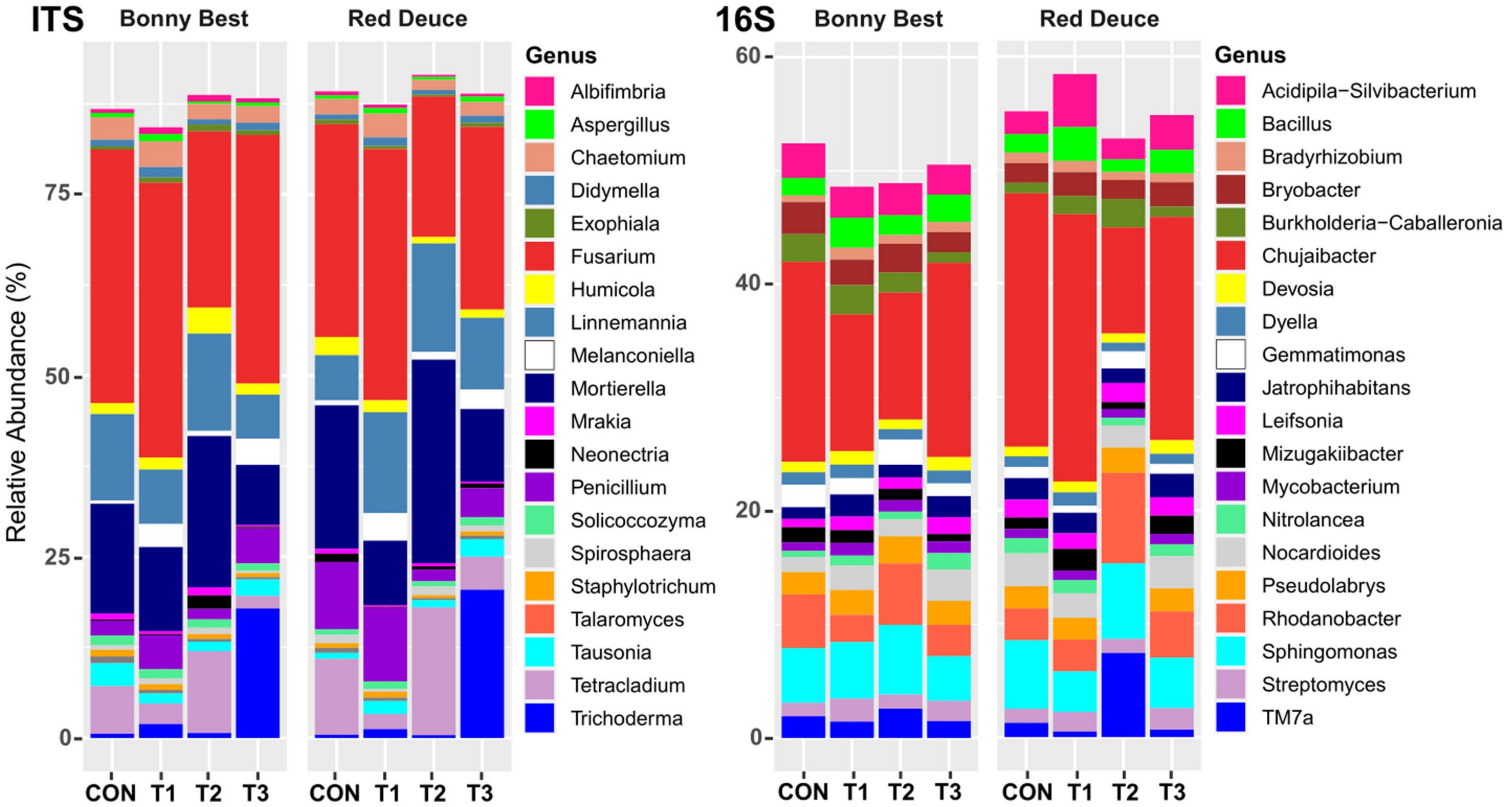
Effect of *Trichoderma* treatment on the mean relative abundance of the top 20 fungal and bacterial genera. Treatments include CON (control treatment), T1 (pre-transplant treatment with *T. virens*), T2 (at-transplant treatment with *T. virens*), and T3 (post-transplant treatment with *T. virens*).

At the genus level, among all treatments, T1 resulted in the highest relative abundance of the fungal genera *Albifimbria*, *Aspergillus*, *Chaetomium*, *Fusarium*, *Melanconiella*, and *Penicillium* in the rhizosphere of both varieties (Figure 4). T2 resulted in the highest abundance of *Linnemannia*, *Mortierella*, and *Tetracladium* and the lowest abundance of *Fusarium* in both varieties. For bacterial genera, compared to the other treatments, T2 increased the abundance of *Gemmatimonas*, *Pseudolabrys*, *Rhodanobacter*, *Sphingomonas*, and *TM7a* but decreased the abundance of *Chujaibacter*, *Devosia*, *Dyella*, and *Jatrophihabitans* in both varieties (Figure 4).

### Effect of *Trichoderma* treatment and tomato variety on the proportional abundance of specific species, genera, and guilds

**Figure 5:**
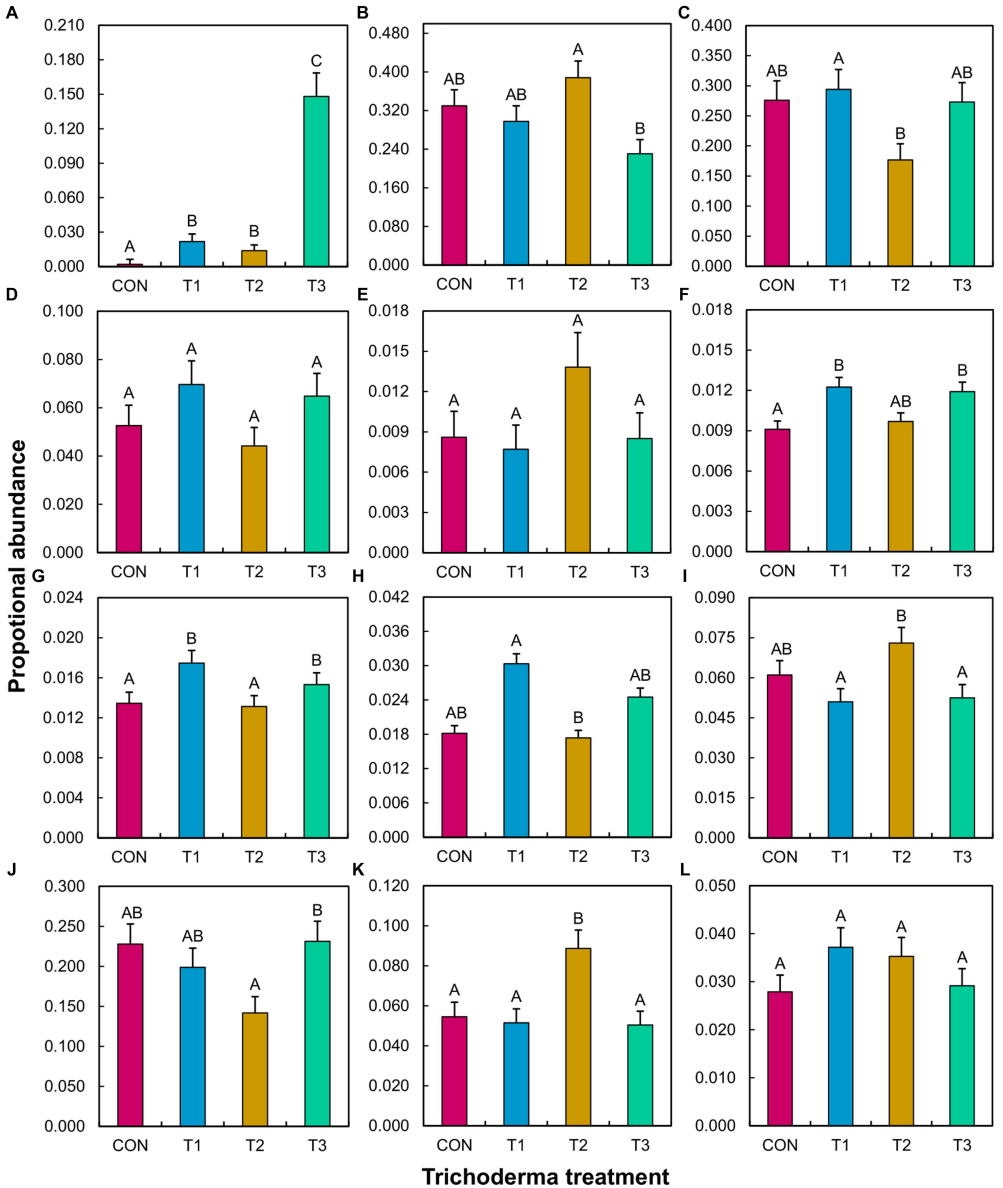
Effect of different *Trichoderma* treatments on the proportional abundance of a specific species, genus, or guild in the rhizosphere across tomato varieties (Bonny Best and Red Deuce). Treatments include CON (control treatment), T1 (pre-transplant treatment with *T. virens*), T2 (at-transplant treatment with *T. virens*), and T3 (post-transplant treatment with *T. virens*). Proportional abundance is the ratio between the read counts of a specific species/genus/guild in a sample and the total read count of the same sample. A guild is a combination of genera that perform a specific ecological role. **(A)** *T. virens*; **(B)** Beneficial fungi (*Acremonium*, *Cadophora*, *Chaetomium*, *Clonostachys*, *Mortierella*, *Paraphaeosphaeria*, *Penicillium*, *Syncephalis*, *Humicola*, *Marquandomyces*, *Metacordyceps*, *Metarhizium*, *Linnemannia*); **(C)** *Fusarium*; **(D)** Non-Fusarial fungal pathogens (*Albifimbria*, *Aspergillus*, *Cladosporium*, *Dactylonectria*, *Didymella*, *Gibellulopsis*, *Melanconiella*, *Microdochium*, *Mycoleptodiscus*, *Neocosmospora*, *Neonectria*, *Paecilomyces*, *Plectosphaerella*, *Pseudopithomyces*, *Rhizoctonia*, *Talaromyces*); **(E)** Biocontrol agents (*Arthrobacter*, *Bdellovibrio*, *Flavisolibacter*, *Lysobacter*, *Massilia*); **(F)** Plant growth promoters (*Devosia*, *Mucilaginibacter*); **(G)** Nitrifiers (*Chujaibacter*, *MND1*, *Nitrolancea*, *Nitrosospira*, *Nitrospira*, *Pseudarthrobacter*, *Pseudolabrys*); **(H)** Symbiotic Nitrogen fixers (*Allorhizobium-Neorhizobium-Pararhizobium-Rhizobium*, *Bradyrhizobium*, *Mesorhizobium*); **(I)** Free-living Nitrogen fixers (*Bacillus*, *Clostridium*, *Paenibacillus*, *Rhodopseudomonas*); **(J)** soil chemicals/toxins detoxifiers (*Mycobacterium*, *Sphingomonas*); **(K)** Denitrifiers (*Burkholderia-Caballeronia-Paraburkholderia*, *Conexibacter*, *Rhodanobacter*); **(L)** Pathogens (*Burkholderia-Caballeronia-Paraburkholderia*, *Ralstonia*, *Streptomyces*). **(E–L)** represent bacteria. Per ANOVA (Supplementary Table S4), the main effect of *Trichoderma* treatment is depicted. Means followed by a common letter are not significantly different. The designation of the significant mean difference between the four levels of the factor “*Trichoderma* treatment” is based on the *p*-values that are adjusted for multiple comparisons using the Tukey–Kramer test at the 5% level of significance (=5% experiment-wise error rate). Error bars represent standard errors.

ANOVA did not reveal a significant *Trichoderma* treatment × tomato variety interaction effect on the proportional abundance of any tested species, genera, or guilds (Supplementary Table S4). The main effect of *Trichoderma* treatment was significant in many cases, except for fungal pathogens other than *Fusarium*, bacterial biocontrol agents, and bacterial pathogens (Supplementary Table S4). The mean separation showed that the abundance of *T. virens* was higher in T3 than in the other treatments (Figure 5). T1 resulted in a significantly higher abundance of *Fusarium* compared to T2, bacterial plant growth promoters compared to CON, nitrifiers compared to CON and T2, and symbiotic nitrogen fixers compared to T2. T1 significantly decreased the abundance of free-living nitrogen fixers and denitrifiers compared to T2. T2 significantly increased the abundance of beneficial fungi compared to T3, free-living nitrogen fixers compared to T1 and T3, and denitrifiers compared to CON, T1, and T3. T2 significantly decreased the abundance of nitrifiers compared to T1 and T3 and soil chemicals/toxin detoxifiers compared to T3.

### Effects of volatiles emitted by cultured rhizomicrobial communities from different *Trichoderma* treatments on the growth of *T. virens* and *F. oxysporum*

**Figure 6:**
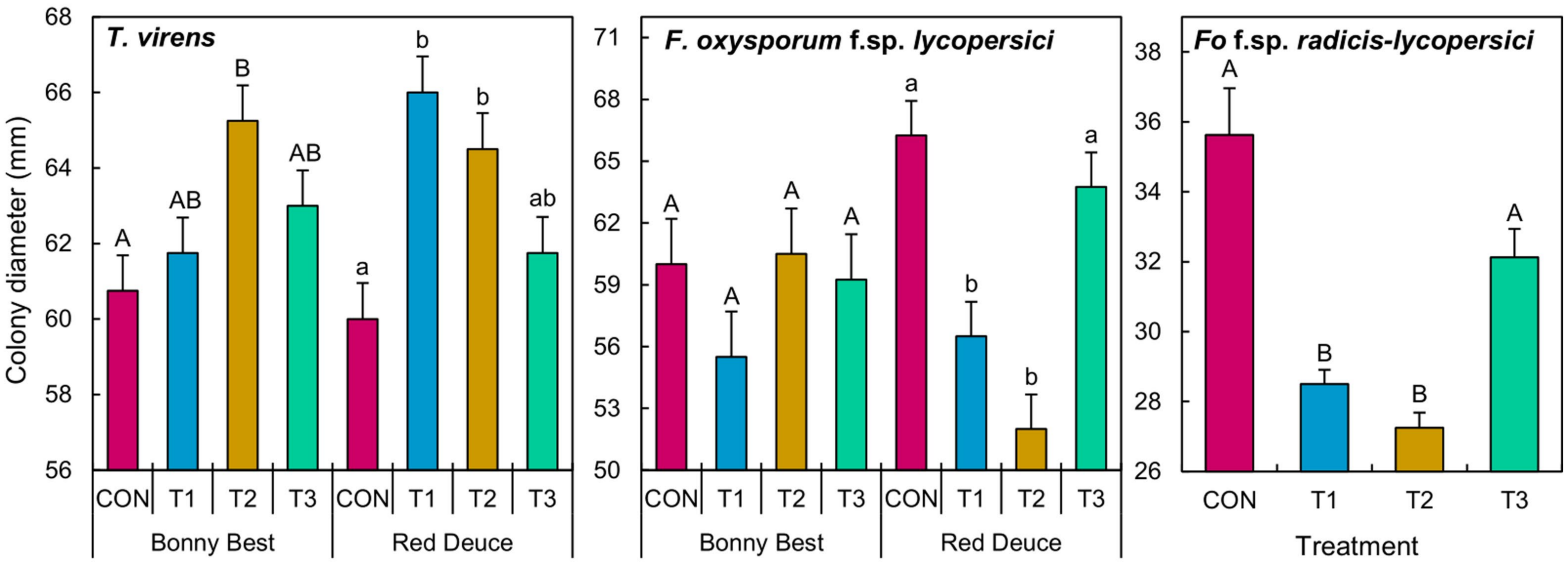
Effect of the volatiles emitted by the cultured fraction of the rhizomicrobiomes isolated from two tomato varieties (Bonny Best and Red Deuce) following *Trichoderma* treatments on the mycelial growth of *T. virens*, *F. oxysporum* f. sp. *lycopersici*, and *F. oxysporum* f. sp. *radicis-lycopersici*. Treatments include CON (control treatment), T1 (pre-transplant treatment with *T. virens*), T2 (at-transplant treatment with *T. virens*), and T3 (post-transplant treatment with *T. virens*). Means followed by the same letters within each letter type (upper or lower case) are not significantly different at the 5% level (=5% experimental-wise error rate) of significance after adjusting the *p*-values for multiple comparisons using the Tukey test. Error bars represent standard errors. In the case of *F. oxysporum* f. sp. *radicis-lycopersici*, the main effect of *Trichoderma* treatment is depicted across two tomato varieties.

A significant *Trichoderma* treatment × tomato variety interaction effect on the colony diameter of *T. virens* and *F. oxysporum* f. sp. *lycopersici* was observed (Supplementary Table S5). The main effect of *Trichoderma* treatment was significant on the growth of *F. oxysporum* f. sp. *radicis-lycopersici*. According to the mean separation, volatiles released by the T2 rhizomicrobiome of Bonny Best increased the growth of *T. virens* compared to CON (Figure 6). In Red Deuce, T1 and T2 rhizomicrobiomes increased the growth of *T. virens* compared to CON. In Bonny Best, *F. oxysporum* f. sp. *lycopersici* growth was not significantly different among the four treatments (Figure 6). However, in Red Deuce, volatiles released by T1 and T2 rhizomicrobiomes significantly reduced the growth of *F. oxysporum* f. sp. *lycopersici* compared to CON and T3 (Figure 6). T1 and T2 rhizomicrobiomes significantly reduced the growth of *F. oxysporum* f. sp. *radicis-lycopersici* compared to CON and T3 across both varieties (Figure 6). Representative colonies of the tested fungi after each treatment are shown in Supplementary Figure S3.

### Effects of water-soluble rhizosphere metabolites on the germination of *T. virens* and *F. oxysporum* conidia

**Figure 7:**
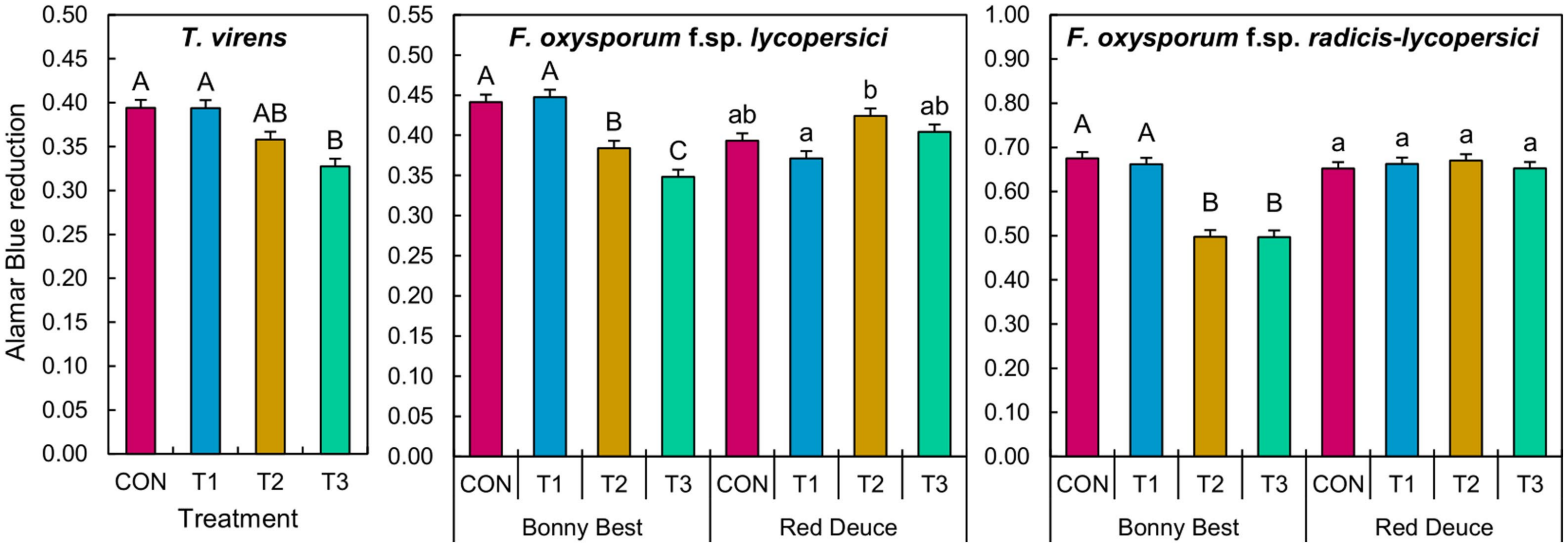
Effect of the water-soluble metabolites extracted from rhizosphere soils of two tomato varieties (Bonny Best and Red Deuce) following *Trichoderma* treatments on the germination of *T. virens*, *F. oxysporum* f. sp. *lycopersici*, and *F. oxysporum* f. sp. *radicis-lycopersici* conidia. The level of Almar Blue reduction is directly proportional to the conidial germination rate. Treatments include CON (control treatment), T1 (pre-transplant treatment with *T. virens*), T2 (at-transplant treatment with *T. virens*), and T3 (post-transplant treatment with *T. virens*). Means followed by the same letters within each letter type (upper or lower case) are not significantly different at the 5% level (=5% experimental-wise error rate) of significance after adjusting the *p*-values for multiple comparisons using the Tukey test. Error bars represent standard errors.

The main effect of *Trichoderma* treatment was significant on the germination of *T. virens* conidia (Supplementary Table S6). A significant *Trichoderma* treatment × tomato variety interaction effect was observed for *F. oxysporum* f. sp. *lycopersici* and *F. oxysporum* f. sp. *radicis-lycopersici* (Supplementary Table S6). According to the mean separation, metabolites from T3 significantly reduced the conidial germination of *T. virens* compared to those of CON and T1 across tomato varieties (Figure 7). T2 and T3 metabolites from Bonny Best reduced the germination of both *F. oxysporum* strains compared to CON and T1 (Figure 7). T2 metabolites from Red Deuce reduced the germination of *F. oxysporum* f. sp. *lycopersici* compared to T1 (Figure 7). No significant germination difference in *F. oxysporum* f. sp. *radicis-lycopersici* was observed among the four treatments in Red Deuce (Figure 7).

## Discussion

### The at-transplant application of *T. virens* enhances tomato growth the most

Our findings showed that the maximum shoot growth of both tomato varieties can be achieved by applying *T. virens* just before transplanting the seedlings. Although the at- and post-transplant applications were equally effective at enhancing tomato root growth and root/shoot ratio, this effect was only significant for Red Deuce. As the tomato variety influences the beneficial effect of *Trichoderma* (Tucci et al., 2011), the effect of its application method on tomato growth could also be influenced by the tomato variety. Evaluation of how the growth of diverse tomato varieties responds to the tested application strategies with more *Trichoderma* spp. and soil types is needed to determine which strategy works best for individual production systems and conditions. Tomato-growing soils likely harbor a variety of pathogens. Since we did not introduce any soilborne tomato pathogen(s) to the soil, an evaluation of how different application methods affect crop growth and health in the presence of known pathogens is also needed. Previous studies showed that *Trichoderma* did not enhance plant growth in the absence of *F. oxysporum* f. sp. *lycopersici* (Ghazalibiglar et al., 2016; Natsiopoulos et al., 2022).

### Greater rhizosphere abundance of *T. virens* does not necessarily result in better tomato growth

The post-transplant application resulted in the highest rhizosphere abundance of *T. virens* in both tomato varieties. Although its abundance was substantially different between the at- and post-transplant applications, we did not observe a significant difference in the growth of both varieties between the two treatments. On the other hand, although the abundance following the at-transplant application was lower than that after the pre-transplant application, the former enhanced tomato growth better. These observations suggested that rhizosphere abundance may not be the primary factor determining the tomato growth-promoting ability of *T. virens*. In this experiment, we introduced *T. virens* inoculum in the form of conidia. Although the presence of conidia is important, how well they germinate and grow determines the success of root colonization and, in turn, the level of growth promotion. Previous studies have shown that *Trichoderma* must colonize plant roots to stimulate plant growth (Kleifeld and Chet, 1992; Olson and Benson, 2007), and that greater colonization results in a greater level of growth promotion (Eslahi et al., 2020). Therefore, although post-transplant application resulted in greater rhizosphere *T. virens* abundance (sequence read counts), it is possible that intact conidia in the rhizosphere, rather than mycelia, served as the main source of DNA contributing to this greater abundance/read count. In fact, the Alamar Blue assay showed that rhizosphere metabolites from post-transplant application significantly reduced the germination of *T. virens* conidia compared to those from control (see below). Additional experiments are needed to determine how different application strategies affect the rhizoplane and root endosphere abundance of *T. virens* and to unravel their possible association with tomato growth in the absence and presence of known soilborne pathogens.

### Changes in the abundance of specific microbial genera and guilds caused by the at-transplant application may contribute to the enhancement of tomato growth

A previous study showed that both bacterial inoculants and inoculant-driven rhizosphere bacterial community shifts contribute to the early growth stimulation of tomato plants (Eltlbany et al., 2019). Soil application of *T. guizhouense* significantly increased cabbage plant biomass, and this effect was attributed to changes in the composition of the resident fungal community, including the increased abundance and number of resident plant growth-promoting fungal taxa (Wang et al., 2023). Our results showed that the at-transplant application of *T. virens* increased the abundance of potentially beneficial fungi (e.g., crop growth promoters, entomopathogenic and nematophagous fungi, mycoparasites) and bacterial biocontrol agents. Such changes may contribute to enhanced tomato growth. In addition, the at-transplant application decreased the abundance of rhizosphere fusaria, including *F. fujikuroi*, *F. graminearum*, *F. neocosmosporiellum*, *F. nurragi*, *F. proliferatum*, *F. sporotrichioides*, *F. equiseti*, and *F. sacchari*. Although they are not known to cause soilborne diseases in tomatoes, their opportunistic pathogenicity and asymptomatic growth hindrance cannot be ruled out. Therefore, the reduction in the abundance of potentially pathogenic fusaria may have helped enhance tomato growth. The increase in free-living nitrogen fixers (predominantly *Bacillus* species) could contribute to increased vegetative growth by enhancing nitrogen availability. A previous study showed that certain *Bacillus* species enhance tomato growth by improving nitrogen fixation and uptake (Masood et al., 2020). Of course, the at-transplant application-mediated increase in denitrifiers may reduce the available nitrogen for tomatoes. Further studies are needed to determine whether *T. virens* application-mediated changes in bacterial taxa related to the nitrogen cycle can significantly affect the overall nitrogen utilization efficiency of tomatoes.

### *T. virens* treatments do not significantly alter the global rhizosphere fungal/bacterial community composition

Competitive displacement of non-target soil microbes and toxicity are key unintended impacts associated with using BCAs like *Trichoderma* (Cook et al., 1996; Brimner and Boland, 2003). Such potentially negative effects on resident soil microbiomes have become a concern (Brimner and Boland, 2003; Szczepaniak et al., 2015). Some microbiome studies provide evidence of *Trichoderma*-driven competitive displacement and significant soil microbial community shifts, whereas others do not. *T. viride* T23 induced significant shifts in the richness, structure, and composition of the rhizosphere fungal and bacterial communities of muskmelon (Zhang et al., 2023). *T. koningiopsis* application similarly shifted both communities in the *Pinus massoniana* rhizosphere (Yu and Luo, 2020). In contrast, *Trichoderma* did not affect the rhizosphere fungal community composition of lettuce (Bellini et al., 2023), the bacterial community composition of tomato (Eltlbany et al., 2019), or the fungal/bacterial community composition of zucchini (Cucu et al., 2020). Therefore, the degree to which *Trichoderma* application affects the non-target rhizosphere microbial community appears to be determined by multiple factors, such as *Trichoderma* species, plants, and soil sources. In the present study, none of the *Trichoderma* application methods significantly decreased the tomato rhizosphere’s fungal/bacterial richness, evenness, or diversity compared to CON. Furthermore, none of the application methods significantly altered the fungal and bacterial community composition of the field soil employed. Therefore, the pre-, at-, and post-transplant applications of *T. virens* did not appear to pose a threat to the rhizomicrobiome structure and function of the tested varieties. New experiments using different soils and tomato varieties are needed to further evaluate this neutral effect.

### Application methods differentially affect mycelial growth and conidial germination of *T. virens* and *F. oxysporum* via rhizomicrobial volatiles and water-soluble soil metabolites

Several sequence data-based studies have suggested that *Trichoderma*-mediated changes in the rhizomicrobiome contribute to soilborne disease suppression. The control of damping-off caused by *F. oxysporum* in *Pinus massoniana* seedlings by *T. koningiopsis* was partly attributed to rhizosphere microbiome shifts (Yu and Luo, 2020). *T. harzianum*-mediated shifts in the maize rhizosphere microbiome contributed to suppressing Fusarium Stalk rot caused by *F. graminearum* (Saravanakumar et al., 2017). In these cases, disease suppression was attributed to an increase in beneficial microbial taxa such as resident rhizosphere biocontrol agents and plant growth promoters. Nonetheless, the mechanisms by which altered microbial communities confer better pathogen control are poorly understood. Microbial volatiles have been shown to play a key role in inter-microbial communication, and some microbes use them to suppress others. For example, *Trichoderma* was shown to use its volatile compounds to suppress *F. oxysporum* (Li et al., 2018). Therefore, extending the role of volatiles in microbe-microbe to microbiome–microbe interactions, we hypothesized that volatiles emitted by the culturable fraction of altered rhizosphere microbes upon different *T. virens* treatments differentially modulate the growth of *T. virens* and two *F. oxysporum* strains. We further hypothesized that water-soluble metabolites extracted from the rhizosphere soil of the two tomato varieties after four *Trichoderma* treatments differentially affect the germination of *T. virens* and *F. oxysporum* conidia. Although none of the *Trichoderma* treatments significantly altered the rhizosphere fungal and bacterial community composition at the global level, they altered the abundance of some major phyla and genera. These alterations can result in altered microbial volatile and rhizosphere metabolite profiles, resulting in differential effects on *Trichoderma* and *F. oxysporum* growth.

Our findings showed that the volatiles emitted by the rhizomicrobial community from both tomato varieties after the at-transplant application better promoted the *in-vitro* mycelial growth of *T. virens*. As discussed above, this may play a direct role in enhancing its root colonization, thus better promoting tomato growth. The same was observed with the pre-transplant application of Red Deuce. On the other hand, the post-transplant application did not cause volatile-mediated mycelial growth enhancement of *T. virens* in both varieties. These findings suggest that the effectiveness of *T. virens* application methods in enhancing its mycelial proliferation by altering rhizomicrobial volatiles is tomato variety-dependent. On the other hand, as revealed by the non-significant *Trichoderma* treatment × tomato variety interaction, the effect of treatment on the rhizosphere metabolite-mediated conidia germination of *T. virens* was not tomato variety-dependent. For instance, compared to the control, soil metabolites from the post-transplant application reduced conidial germination across varieties. Taken together, volatiles and water-soluble metabolites from a given application method do not appear to have similar effects (i.e., synergistic, antagonistic, or neutral) on the modulation of *T. virens* conidial germination and mycelial growth. A reciprocal experiment (effects of volatiles on conidial germination and water-soluble metabolites on mycelial growth) and simultaneous exposure (effects of volatiles and water-soluble metabolites on conidial germination and subsequent mycelial growth) could provide more mechanistic insights.

The volatiles emitted by culturable rhizomicrobial communities of both tomato varieties after the pre- and at-transplant applications significantly suppressed the mycelial growth of *F. oxysporum* f. sp. *radicis-lycopersici*. The same was true for *F. oxysporum* f. sp. *lycopersici*, but only for Red Duce. The post-transplant application had a non-significant effect on the growth of both *Fusarium* species. Metabolites from Bonny Best after the at- and post-transplant applications significantly suppressed the conidial germination of both *F. oxysporum* strains. Taken together, these results suggest that the application method matters for building suppressiveness against *Fusarium* pathogens. The importance of application methods was previously reported; for example, suppressiveness against pre-emergence damping-off of pea caused by *Pythium* spp. was only achieved by planting *Trichoderma* conidia-treated seeds but not by introducing them into the conducive soil (Harman et al., 1980). In contrast, suppressiveness against *Rhizoctonia solani* was induced by both seed and soil applications (Harman et al., 1980). Our results further suggested that *Trichoderma* application-mediated changes in both the rhizosphere metabolite and rhizomicrobial emitted volatile profiles may be important in suppressing *F. oxysporum* in susceptible tomato varieties such as Bonny Best, while volatiles are more important for resistant varieties such as Red Deuce.

The findings of our *in-vitro* assays, in general, suggest that some of the tested *T. virens* application methods could build rhizomicrobiome-mediated mycelial growth suppressiveness against some *Fusarium* pathogens in a tomato variety-independent manner, while the same for some other pathogens can be tomato variety-dependent. It would be vital to redo the experiment by introducing pathogens to the soil used in our experiments, growing the same tomato, and quantifying the actual pathogen levels in the rhizosphere to see if different *T. virens* application methods truly build suppressiveness against the pathogens. It is also important to investigate the durability of such microbial ecology-based pathogen suppressiveness by repeating the aforementioned experiment over multiple tomato growth cycles (with and without applying a new *Trichoderma* inoculant). Furthermore, as the influence of soil source on the outcomes described above cannot be neglected, expanded investigations are required to test whether different soils, *Trichoderma* species, application methods, and tomato varieties in combination similarly modulate the rhizosphere suppressiveness against soilborne pathogens via rhizomicrobial volatiles and water-soluble metabolites.

## Conclusion

The at-transplant application of *T. virens* promoted tomato growth better than the pre- and post-transplant applications. In commercial tomato production, tomato seedlings are typically raised using a dual-tray system (starter and bottom trays). Therefore, the method we used to introduce *Trichoderma* can be readily incorporated into commercial systems to enhance the vigor and health of tomatoes. Our findings showed that *T. virens* (particularly through the at-transplant application) could potentially be used to build rhizomicrobiome-based suppressiveness against soilborne pathogens like *F. oxysporum*. However, further experiments with more explanatory variables are required before implementing this method for tomato production. Specific questions include whether different *Trichoderma* species work similarly, whether different tomato varieties and soil types result in similar tomato growth and rhizomicrobiome responses to *Trichoderma* treatments, whether rhizomicrobiome changes driven by *Trichoderma* treatments contribute to suppressing other soilborne pathogens, and whether the assays used to evaluate the effects of volatile and water-soluble soil metabolites are reliable indicators for predicting the (a) conduciveness/suppressiveness of diverse field soils for *Trichoderma* proliferation and (b) *Trichoderma*-mediated soil conduciveness/suppressiveness against diverse pathogens.

## Data availability statement

The datasets presented in this study can be found in online repositories. The names of the repository/repositories and accession number(s) can be found in the article/Supplementary material.

## Author contributions

AB: Conceptualization, Data curation, Formal analysis, Funding acquisition, Investigation, Methodology, Software, Validation, Visualization, Writing – original draft, Writing – review & editing. SK: Conceptualization, Funding acquisition, Project administration, Resources, Supervision, Writing – review & editing.
