# Supplementary material for: Trichoderma application methods differentially affect the tomato growth, rhizomicrobiome, and rhizosphere soil suppressiveness against Fusarium oxysporum
Source: Front Microbiol. 2024 Feb 27;15:1366690. doi: 10.3389/fmicb.2024.1366690 (PMC10929717; doi:10.3389/fmicb.2024.1366690)
Supplement: Supplementary file 1 [file Data_Sheet_1.pdf]

Supplementary document

Trichoderma application methods differentially affect the tomato growth, rhizomicrobiome, and rhizosphere soil suppressiveness against *Fusarium oxysporum*

Authors: Ananda Y. Bandara, Seogchan Kang

Affiliation: Department of Plant Pathology & Environmental Microbiology, The Pennsylvania State University, University Park, PA 16802, USA.

Corresponding author: Seogchan Kang

Email: Ananda Y. Bandara - axb1739@psu.edu; Seogchan Kang - sxk55@psu.edu

ORCID: Ananda Y. Bandara: 0000-0003-0725-8226
Seogchan Kang: 0000-0003-2291-5634

The raw sequence data generated for this study are available in the **NCBI SRA: PRJNA1060682**

Supplementary Table S1. *P* values from analysis of variance showing the effect of tomato variety, *Trichoderma* treatment, and their interaction on three growth indicators. Treatments include CON (control treatment), T1 (pre-transplant treatment with *T. virens*), T2 (at-transplant treatment with *T. virens*), and T3 (post-transplant treatment with *T. virens*). Tested tomato varieties include Bonny Best and Red Deuce.

Variable	Effect		
	Variety (V)	Treatment (T)	V × T
Shoot weight	<0.0001	0.0013	0.0723
Root weight	0.0578	0.0012	0.0037
Root: Shoot ratio	0.6953	0.0013	0.0121

Supplementary Table S2. *P* values from analysis of variance showing the effect of tomato variety, *Trichoderma* treatment, and their interaction on different Alpha diversity indicators related to rhizosphere fungal and bacterial communities. Treatments include CON (control treatment), T1 (pre-transplant treatment with *T. virens*), T2 (at-transplant treatment with *T. virens*), and T3 (post-transplant treatment with *T. virens*). Tested tomato varieties include Bonny Best and Red Deuce.

Indicator	Community	Effect		
		Variety (V)	Treatment (T)	V× T
Chao 1	Fungi	0.0736	0.2286	0.9199
	Bacteria	0.0094	0.2317	0.2495
Coverage	Fungi	0.1813	0.9583	0.6149
	Bacteria	0.0274	0.3451	0.3152
Shannon	Fungi	0.0002	0.0164	0.0005
	Bacteria	0.0018	0.0331	0.0215
Pielou	Fungi	0.0018	0.0503	0.0355
	Bacteria	0.0273	0.5178	0.2349
Core abundance	Fungi	0.0401	<0.0001	0.1587
	Bacteria	0.0571	0.3949	0.5409
Rare abundance	Fungi	0.1602	<0.0001	0.1142
	Bacteria	0.0685	0.2276	0.5890

Supplementary Table S3. Correlation of determination (R^2) and adjusted *P* values from the pairwise permutational multivariate analysis of variance (PERMANOVA) and pairwise beta dispersion tests for different *Trichoderma* treatment pairs related to rhizosphere fungal and bacterial communities across two tomato varieties. Treatments include CON (control treatment), T1 (pre-transplant treatment with *T. virens*), T2 (at-transplant treatment with *T. virens*), and T3 (post-transplant treatment with *T. virens*). Tested tomato varieties include Bonny Best and Red Deuce.

Pair	Fungi			Bacteria		
	PERMANOVA		Beta dispersion	PERMANOVA		Beta dispersion
	R^2	*P*. adjusted	*P*. adjusted	R^2	*P*. adjusted	*P*. adjusted
T1 vs T2	0.147	0.06	0.289	0.116	0.090	0.616
T1 vs T3	0.047	1	0.351	0.056	1	0.104
T1 vs CON	0.048	1	0.584	0.071	0.750	0.379
T2 vs T3	0.131	0.018	0.868	0.152	0.018	0.106
T2 vs CON	0.082	0.288	0.533	0.090	0.204	0.559
T3 vs CON	0.060	0.702	0.631	0.065	0.594	0.193

Supplementary Table S4. *P* values from analysis of variance showing the effect of tomato variety, *Trichoderma* treatment, and their interaction on the proportional abundance of a specific species, genus, or guild in the rhizosphere. Proportional abundance is the ratio between the read counts of a specific species/genus/guild (noted under the table) in a sample and the total read count of the same sample. A guild is a combination of genera that can perform a specific ecological role. Treatments include CON (control treatment), T1 (pre-transplant treatment with *T. virens*), T2 (at-transplant treatment with *T. virens*), and T3 (post-transplant treatment with *T. virens*). Tested tomato varieties include Bonny Best and Red Deuce.

Kingdom	Species/Genus/Guild	Effect		
		Variety (V)	Treatment (T)	V × T
Fungi	*T. virens*	0.6367	<0.0001	0.9979
	Beneficial[1]	0.1896	0.0130	0.9369
	Fusaria	0.0614	0.0446	0.9508
	Non-Fusarium pathogens[2]	0.2630	0.1676	0.6279
Bacteria	Biocontrol agents[3]	0.2830	0.1503	0.1655
	Plant growth promoters[4]	0.2108	0.0029	0.2794
	Nitrifiers[5]	0.1172	0.0367	0.2627
	Symbiotic N-fixers[6]	0.5103	0.0424	0.2334
	Free-lining N-fixers[7]	0.2905	<0.0001	0.1327
	Soil toxin degraders[8]	0.8732	0.0201	0.2261
	Pathogens[9]	0.3727	0.2365	0.2015
	Denitrifiers[10]	0.7236	0.0025	0.0882

[1]*Acremonium*, *Cadophora*, *Chaetomium*, *Clonostachys*, *Mortierella*, *Paraphaeosphaeria*, *Penicillium*, *Syncephalis*, *Humicola*, *Marquandomyces*, *Metacordyceps*, *Metarhizium*, *Linnemannia*.
[2]*Albifimbria*, *Aspergillus*, *Cladosporium*, *Dactylonectria*, *Didymella*, *Gibellulopsis*, *Melanconiella*, *Microdochium*, *Mycoleptodiscus*, *Neocosmospora*, *Neonectria*, *Paecilomyces*, *Plectosphaerella*, *Pseudopithomyces*, *Rhizoctonia*, *Talaromyces*.
[3]*Arthrobacter*, *Bdellovibrio*, *Flavisolibacter*, *Lysobacter*, *Massilia*.
[4]*Devosia*, *Mucilaginibacter*.
[5]*Chujaibacter*, *MND1*, *Nitrolancea*, *Nitrosospira*, *Nitrospira*, *Pseudarthrobacter*, *Pseudolabrys*.
[6]*Allorhizobium-Neorhizobium-Pararhizobium-Rhizobium*, *Bradyrhizobium*, *Mesorhizobium*.
[7]*Bacillus*, *Clostridium*, *Paenibacillus*, *Rhodopseudomonas*
[8]*Mycobacterium*, *Sphingomonas*.
[9]*Burkholderia-Caballeronia-Paraburkholderia*, *Ralstonia*, *Streptomyces*.
[10]*Burkholderia-Caballeronia-Paraburkholderia*, *Conexibacter*, *Rhodanobacter*.

Supplementary Table S5. *P* values from analysis of variance showing the effect of tomato variety, *Trichoderma* treatment, and their interaction on the mycelial growth modulation of *T. virens* and *F. oxysporum* cultured on potato dextrose agar by volatile compounds produced by cultured rhizosphere microbes after treatments. Treatments include CON (control treatment), T1 (pre-transplant treatment with *T. virens*), T2 (at-transplant treatment with *T. virens*), and T3 (post-transplant treatment with *T. virens*). Tested tomato varieties include Bonny Best and Red Deuce.

Colony diameter	Effect		
	Variety (V)	Treatment (T)	V × T
Trichoderma virens	0.5805	0.0005	0.0242
Fusarium oxysporum f. sp. *lycopersici*	0.5628	0.0018	0.0049
Fusarium oxysporum f. sp. *radicis-lycopersici*	0.8836	0.0002	0.2135

Supplementary Table S6. *P* values from analysis of variance showing the effect of tomato variety, *Trichoderma* treatment, and their interaction on the modulation of the conidial germination rates (measured via Alamar Blue reduction assay) of *T. virens* and *F. oxysporum* by metabolites extracted from the rhizosphere soils collected after treatments. Treatments include CON (control treatment), T1 (pre-transplant treatment with *T. virens*), T2 (at-transplant treatment with *T. virens*), and T3 (post-transplant treatment with *T. virens*). Tested tomato varieties include Bonny Best and Red Deuce.

Alamar Blue reduction	Effect		
	Variety (V)	Treatment (T)	V × T
Trichoderma virens	<0.0001	0.0002	0.0797
Fusarium oxysporum f. sp. *lycopersici*	0.3265	0.0004	<0.0001
Fusarium oxysporum f. sp. *radicis-lycopersici*	<0.0001	<0.0001	<0.0001

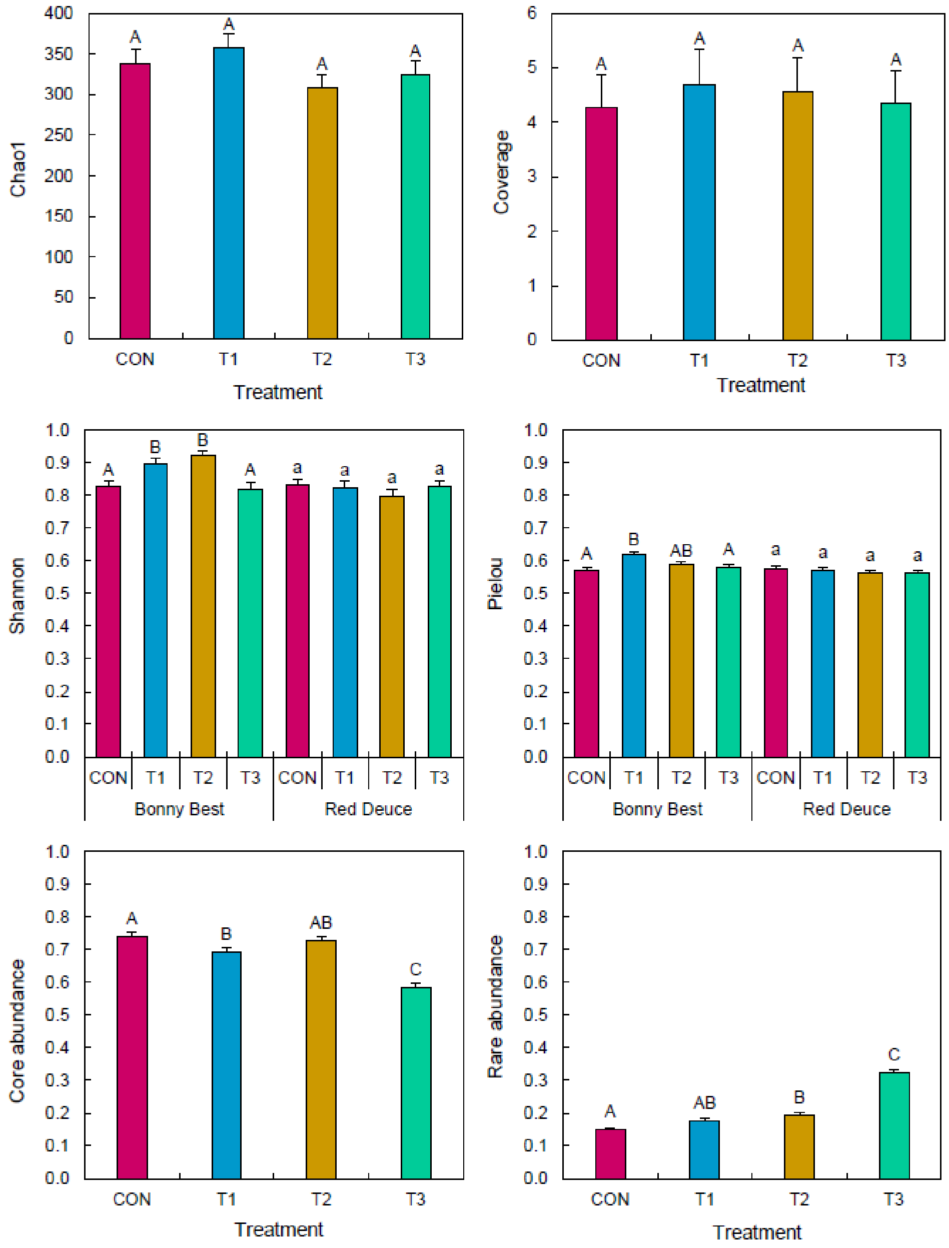

Supplementary Figure S1. Effects of four *Trichoderma* treatments and two tomato varieties on the mean fungal α-diversity indicators. Treatments include CON (control treatment), T1 (pre-transplant treatment with *T. virens*), T2 (at-transplant treatment with *T. virens*), and T3 (post-transplant treatment with *T. virens*). Tested tomato varieties include Bonny Best and Red Deuce. Means followed by a common letter within each letter type (upper or lower case) are not significantly different. The designation of the significant mean difference between the four levels of the factor *Trichoderma* treatment is based on the p-values that are adjusted for multiple comparisons using the Tukey-Kramer test at the 5% level of significance (= 5% experiment-wise error rate). Error bars represent standard errors. Per ANOVA results (Supplementary Table S2), the simple effect of *Trichoderma* treatment within each level of tomato variety is depicted in the case of Shannon diversity and Pielou evenness, while the main effect of *Trichoderma* treatment is depicted for other alpha diversity indicators.

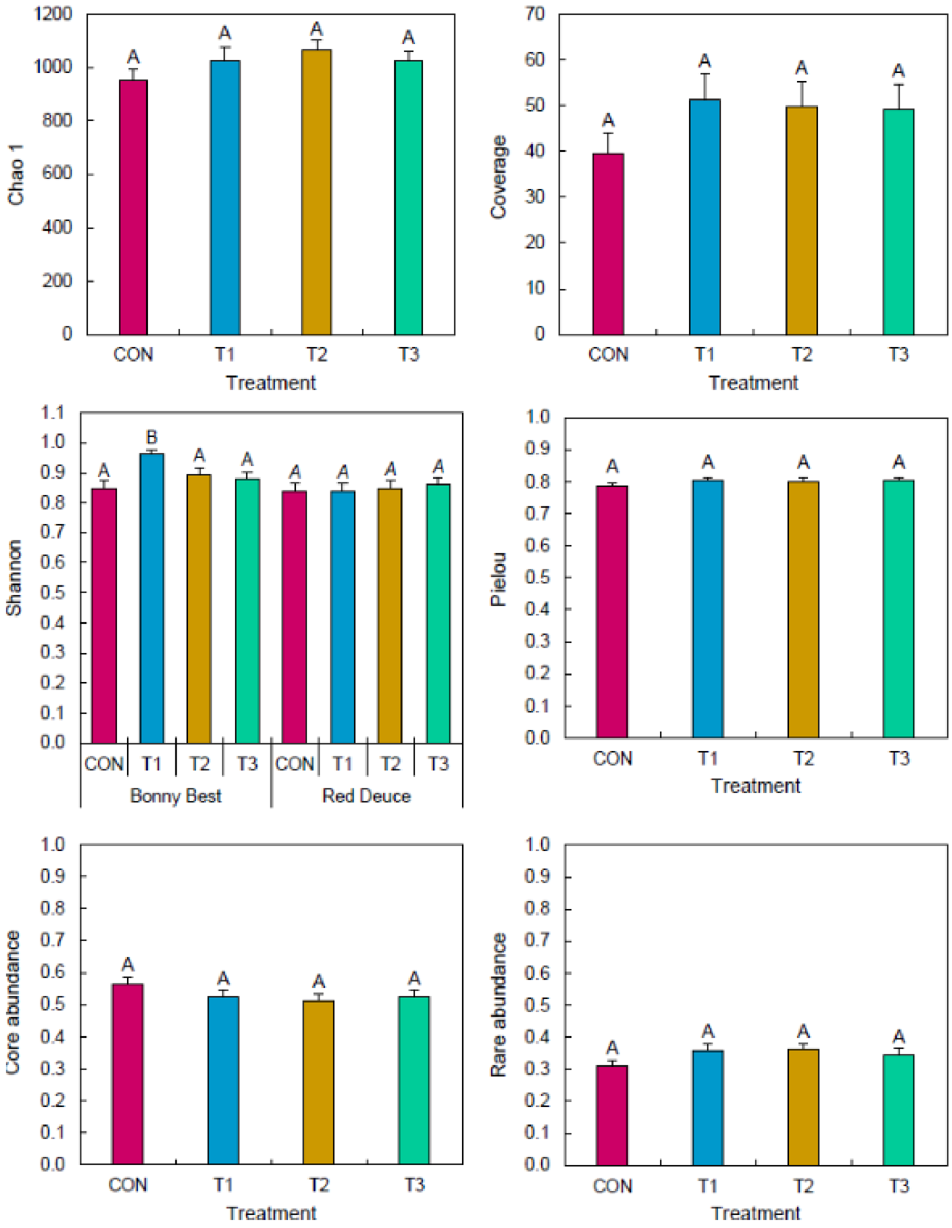

Supplementary Figure S2. Effects of four *Trichoderma* treatments and two tomato varieties on the mean bacterial α-diversity indicators. Treatments include CON (control treatment), T1 (pre-transplant treatment with *T. virens*), T2 (at-transplant treatment with *T. virens*), and T3 (post-transplant treatment with *T. virens*). Tested tomato varieties include Bonny Best and Red Deuce. Means followed by a common letter within each letter type (upper or lower case) are not significantly different. The designation of the significant mean difference between the four levels of the factor *Trichoderma* treatment is based on the p-values that are adjusted for multiple comparisons using the Tukey-Kramer test at the 5% level of significance (= 5% experiment-wise error rate). Error bars represent standard errors. Per ANOVA results (Supplementary Table S2), the simple effect of *Trichoderma* treatment within each level of tomato variety is depicted in the case of Shannon diversity, while the main effect of *Trichoderma* treatment is depicted for other alpha diversity indicators.

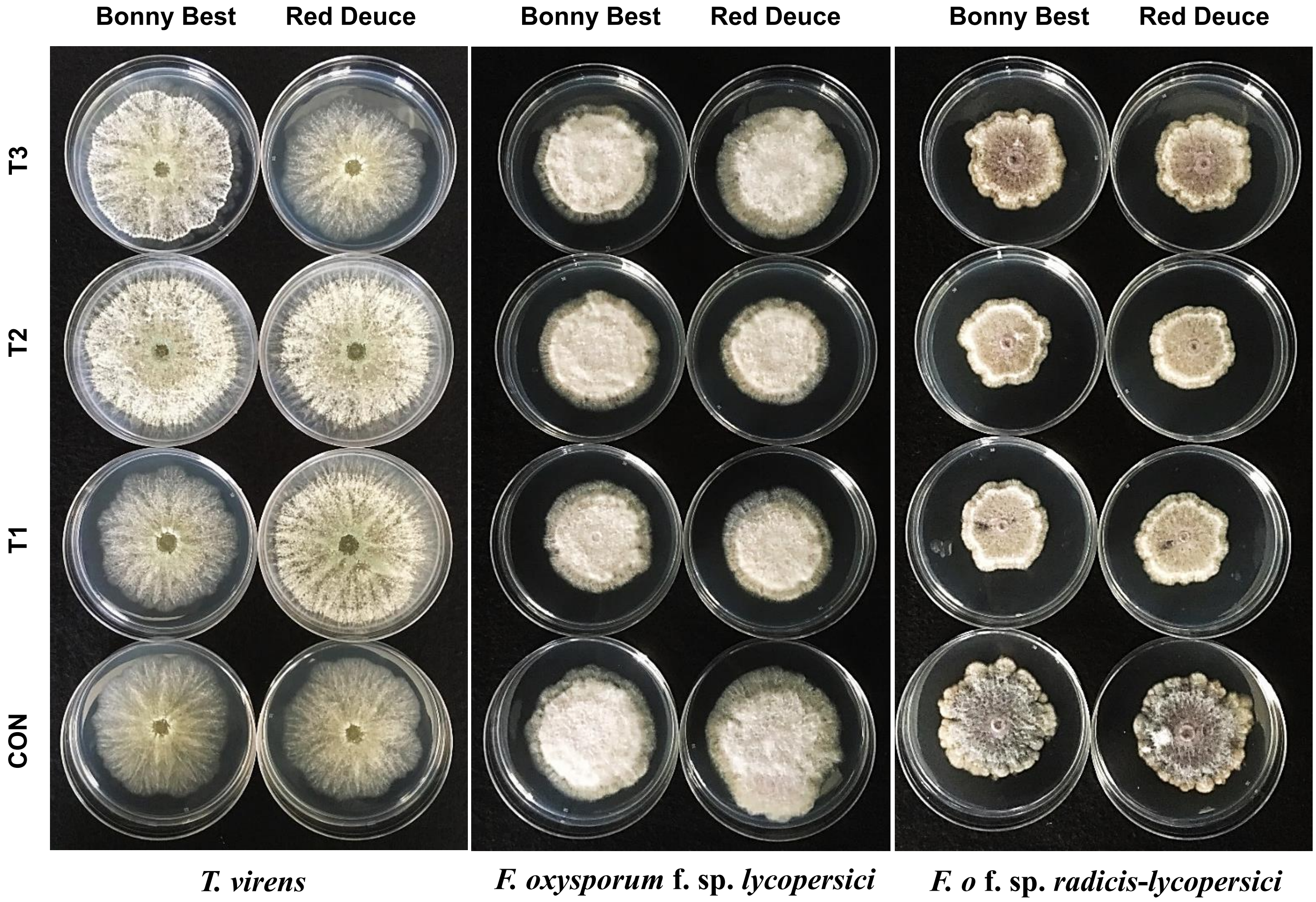

Supplementary Figure S3. Effect of the volatiles emitted by the cultured fraction of rhizomicrobiomes associated with two tomato varieties after different *Trichoderma* treatments on the mycelial growth of tested fungi. Treatments include CON (control treatment), T1 (pre-transplant treatment with *T. virens*), T2 (at-transplant treatment with *T. virens*), and T3 (post-transplant treatment with *T. virens*). Tested tomato varieties include Bonny Best and Red Deuce.
